# Development of a Colic Scoring System to Predict Outcome in Horses

**DOI:** 10.3389/fvets.2021.697589

**Published:** 2021-10-08

**Authors:** Alanna Farrell, Kevin Kersh, Rachel Liepman, Katarzyna A. Dembek

**Affiliations:** ^1^Department of Veterinary Clinical Sciences, College of Veterinary Medicine, Iowa State University, Ames, IA, United States; ^2^Chaparral Veterinary Medical Center, Cave Creek, AZ, United States; ^3^Department of Veterinary Clinical Sciences, College of Veterinary Medicine, North Carolina State University, Raleigh, NC, United States

**Keywords:** horse, colic, survival, prognosis, retrospective

## Abstract

Acute abdominal pain in the horse is a common emergency presenting to equine practices. The wide variety of etiologies makes prognosticating survival a challenge. A retrospective, multi-institutional clinical study was performed to determine clinical parameters associated with survival of horses with colic, and to use them to develop a colic survival scoring system. The scoring system was then validated using clinical data in the prospective portion of the study. Medical records from 67 horses presenting for acute abdominal pain were evaluated to develop the colic assessment score. Twenty eight variables were compared between survivors and non-survivors and entered into logistic regression models for survival. Of these, six variables were included in the colic assessment score. A total colic assessment score range was from 0 to 12, with the highest score representing the lowest probability of survival. The optimal cutoff value to predict survival was seven resulting in an 86% sensitivity and 64% specificity with a positive predictive value of 88% and a negative predictive value of 57%. Data from 95 horses presenting for abdominal pain to two equine hospitals was then collected prospectively to validate the colic assessment score. Horses from the prospective portion of the study that received a score >7 were classified as predicted to die and those with a score ≤7 were predicted to survive. The classification was compared to the actual outcome, of which the sensitivity, specificity, positive and negative predictive values of the colic assessment score were 84, 62, 88, and 52%, respectively.

## Introduction

Acute abdominal pain from gastrointestinal disorders can be successfully treated medically or surgically (1–4). However, treatment can be costly and emotionally tolling for horse owners. Prediction of the likelihood of survival using clinical parameters at presentation would aid clinicians in making important therapeutic decisions. The prediction of whether or not a horse is likely to survive a colic episode is oftentimes based on the veterinarian's clinical impression of the animal. This typically depends on the horse's comfort level at initial evaluation, clinical history, physical exam parameters, rectal examination, peritoneal fluid evaluation, abdominal ultrasound findings, and clinical pathology (5, 6). Together, these clinical findings provide useful information that is essential for prognostication.

A significant portion of the clinical exam for a horse presenting with colic signs is based on human interpretation (comfort level, transrectal palpation, and ultrasound findings) (6). However, it is important to base prognoses and therapies on both empirical evidence and the clinical picture to avoid cognitive biases. Cognitive biases have been shown to contribute to physician diagnostic errors and it is reasonable to presume that veterinarians are not immune to the same biases (7). Creation of a numerical scoring system would aid in making the assessment of the patient more objective and could ensure that more unbiased clinical findings are also considered.

Scoring systems for horses with colic signs or signs of SIRS (systemic inflammatory response syndrome) have been previously evaluated (3, 8). Furr et al. developed a colic severity score in 1995 and used heart rate, peritoneal fluid total protein, blood lactate concentration, and abnormal mucous membranes as predictors of outcome (8). However, the performance of scoring systems varies among populations of horses or over time in a given population. A large retrospective study performed by van der Linden and colleagues in 2003 reported no significant association between survival and packed cell volume or appearance of mucous membranes (9), which is in contrast to the findings in Furr's study. Previous studies have helped to improve the objectivity of pain assessment in colic cases by using pain scoring systems, and these have also demonstrated the predictive value of pain status in colic cases (10–12). One of the primary objectives of our present study was to evaluate a more current population horses with colic, and to determine if there were any other parameters that could be useful in prognosticating survival of colic. We aimed to use these parameters to create a scoring system to help predict prognosis of survival. The second part of this study aimed at validating the scoring system using a prospective population, evaluating horses presenting for abdominal pain at two hospitals over a 1 year period.

## Materials and Methods

**Figure 1:**
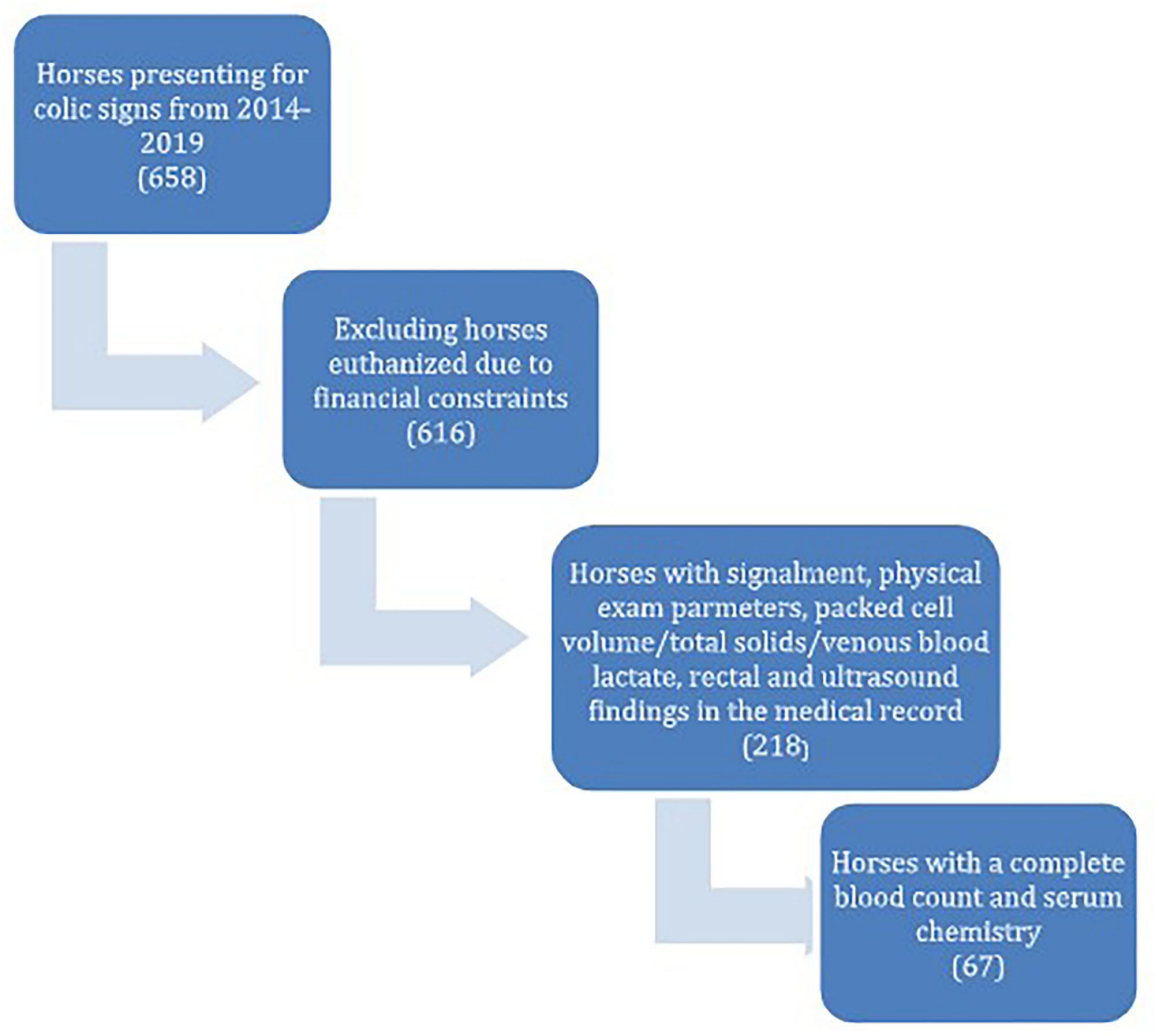
Flow chart depicting case selection for the retrospective population of the study.

The first part of this study was retrospective in nature. The medical records of 658 horses presenting to the Lloyd Veterinary Medical Hospital for signs of colic between the years of 2014–2019 were evaluated. Horses that were euthanized due to financial constraints were excluded (42 horses). Animals younger than 6 months of age were excluded. Colitis cases were excluded. The inclusion criteria required that signalment, physical exam parameters, packed cell volume, total solids, venous blood lactate, transrectal palpation findings, transabdominal ultrasound findings, complete blood count, and serum chemistry values all be present in the medical record. This limited the final population to a total of 67 horses (Figure 1). In total, 28 variables were assessed for each patient. Ten of the variables were chosen based on findings that were typically available in the medical record for horses presenting for colic signs (heart rate, respiratory rate, temperature, capillary refill time, peripheral lactate, abdominal ultrasound findings, transrectal abdominal palpation findings, volume of net gastric reflux, presence of diarrhea, and peritoneal fluid lactate). The values provided by the complete blood count and serum chemistry were included and evaluated in order to determine the predictive value of these variables in regards to survival. All 28 variables were compared between survivors and non-survivors and entered into univariate logistic regression analyses for survival. Survivors were defined as surviving to discharge from the hospital. Blood was drawn from the jugular vein of all patients. A complete blood count was obtained *via* an *Element HT5* (Heska) veterinary hematology analyzer. A serum chemistry was obtained using a *VETSCAN*® VS2 (Abaxis) chemistry analyzer. Lactate was obtained using a *Lactate Plus* (Nova Biomedical) portable analyzer. Six of the 28 variables were included in the colic assessment score (heart rate, respiratory rate, total serum calcium concentration, blood lactate concentration, abnormal ultrasound and rectal findings). These six variables were chosen based on their association with survival. The rectal and abdominal ultrasound findings were classified as dichotomous variables and recorded as “normal” or “abnormal.” Abnormal rectal findings included gas distension, distended loops of small intestine, colon displacement, and impactions (both large and small colon). Normal rectal findings were the absence of any of these findings. Abnormal ultrasound findings included dilated loops of small intestine (>5 cm in diameter), thickened small intestinal wall (>3 mm), increased abdominal fluid, gastric distension (imaged past the 13th rib), dilated colonic vessels, thickened large colon wall (>8 mm) and inability to visualize the left kidney (12–14). Both the rectal and abdominal ultrasound examinations were performed by equine medicine or equine surgery residents in their 1st, 2nd, or 3rd year of training under the supervision of either an ACVIM or ACVS diplomate. The examinations were not standardized across the population because of the retrospective nature of the data.

Data for the prospective study was obtained from horses presenting for acute abdominal pain to either Lloyd Veterinary Medical Center or Chaparral Veterinary Medical Center. Data from 95 horses in total were used in the prospective study. This data was gathered over the course of 1 year. After all of the data had been obtained, the six variables of the colic assessment score (heart rate, respiratory rate, total serum calcium concentration, blood lactate concentration, ultrasound, and rectal findings) were tabulated to determine a final assessment score for each animal retrospectively.

### Statistics

Data sets were tested for normality using the Shapiro-Wilk statistic and variables were not found to be normally distributed. Medians and interquartile ranges were calculated for continuous variables. The Mann-Whitney-U test was used to compare continuous variables between survivor and non-survivor groups in the retrospective study. Relationships between survival and categorical variables were analyzed using contingency tables, chi-square analysis (abnormal rectal exam), and Fisher's Exact test (presence of reflux and diarrhea, abnormal abdominal ultrasound, CRT >2 s. Clinical and laboratory variables that were different between survivors and non-survivors were analyzed in univariate logistic regressions to determine which variables provided the most accurate prediction of survival. Abnormal rectal exam was added to the univariate logistic regression analysis because it was considered an important clinical parameter associated with outcome in horses presented for colic (15). Multiple univariate logistic regression models were applied to calculate odds ratios (OR) and 95% confidence intervals for the outcome. This procedure identified six variables that were included in the final scoring system (Colic Assessment Score-CAS). Data for the variables found to be significant were plotted as histograms with the proportion of survivors and non-survivors at each increment of measurement identified. Cutoff points were established to divide the range of responses for each variable into three different categories based on percentage of non-survivors.

In order to determine the area under the curve (AUC) and a cutoff value above which survival could be most reliably predicted by the CAS, a receiver operating characteristic (ROC) curve was created. Univariate logistic regression analysis for the CAS to predict survival was performed in the prospective study. A commercial statistic software program (IBM SPSS Statistics version 24, IBM corp., NY and Graph Pad Prism version 8, GraphPad Software, CA) was used.

## Results

### Colic Assessment Score (CAS)

After the 28 variables were compared between survivors and non-survivors and entered into univariate logistic regression analyses for survival (Table 1), data for each of the six selected variables (heart and respiratory rate, blood lactate and total calcium concentrations, abnormal abdominal ultrasound and abnormal rectal exam) were plotted as histograms with the proportion of survivors and non-survivors at each increment of measurement identified. Cutoff points were established to divide the range of responses for each variable into three different categories based on low, medium and high percentage of non-survivors. Sub-scores 0, 1 or 2 were assigned to each category. A subscore 0 and 2 were assigned to the category with the lowest and the highest percentage of non-survivors, respectively. Categories and sub-scores were entered into a table that was used to calculate CAS (Table 2).

**Table 1:** Laboratory and clinical variables categorized by outcome in horses presented for colic in the retrospective study (median and interquartile range).

**Variable**	**Survivors**	**Non-survivors**	***P*-value**
	**(*n* = 52)**	**(*n* = 15)**	
Heart rate (bpm)	52 (40–61)	64 (56–79)	0.004
Temperature (F)	99.9 (99–100.8)	99.9 (99.3–101.3)	0.3
Respiratory rate (bpm)	20 (16–30)	31 (24–38)	0.005
PCV (%)	38 (33–42)	41 (33.5–55)	0.2
Total Protein (mg/dL)	7 (6.5–7.6)	6.9 (6–7.5)	0.4
Blood lactate (mmol/L)	0.9 (0–2)	3 (1.2–7.35)	0.008
Peritoneal fluid lactate (mmol/L)	0 (0–3.5)	6.3 (2.9–14.1)	0.001
White blood cell count	7.54 (6–9.8)	5.86 (2.9–11.8)	0.5
Neutrophil count	5 (3.8–7.5)	3.66 (1.3–8)	0.3
Band neutrophil count	0.07 (0–0.2)	0.3 (0.08–0.9)	0.02
Fibrinogen (mg/dL)	300 (300–500)	500 (300–600)	0.03
Sodium (mEq/L)	136 (133–137.3)	133 (129–136.8)	0.07
Potassium (mEq/L)	3.8 (3.4–4)	3.3 (3.2–3.9)	0.2
Chloride (mEq/L)	100 (99–103)	102 (91.7–103)	0.3
Bicarbonate (mEq/L)	28 (26–30)	27 (24–31.5)	0.4
Total calcium (mEq/L)	11.6 (10. −12.05)	10.1 (9–11.5)	0.005
BUN (mg/dL)	19 (15–23.5)	21 (15–25)	0.8
Creatinine (mg/dL)	1.4 (1.1–1.6)	1.5 (1.2–1.8)	0.2
Glucose (mg/dL**)**	113.5 (99.5–131.5)	152 (104–178)	0.14
Albumin (mg/dL)	2.9 (2.5–3.1)	2.7 (2.2–3.2)	0.3
Creatinine Kinase (IU/L)	247 (156.5–672)	241 (120.5–549.5)	0.5
GGT (mg/dL)	30 (24–44)	33 (16.2–57.5)	0.6
Triglycerides (mg/dL)	28 (21–50)	95 (93–115)	0.01
Abnormal rectal exam (%)	40	52	0.2
Abnormal abdominal ultrasound (%)	46	76	0.04
Presence of reflux (%)	23	46	0.11
Presence of diarrhea (%)	6	17	0.2
CRT >2 s	14	17	0.7

**Table 2:** Colic assessment score.

**Variables**	**Scores**			**Sub-score**
	**0**	**1**	**2**	
Heart rate (bpm)	26–45	46–60	≥61	
Respiratory rate (bpm)	5–16	17–28	≥29	
Total Ca (mg/dL)	≥11.9	10.6–11.8	6–10.5	
Blood lactate (mmol/L)	0–2		>2.1	
Abnormal ultrasound	No		Yes	
Abnormal rectal exam	No		Yes	
Total Score				

### Retrospective

The median age of horses from the retrospective study was 10 years (3–16 years; interquartile range) and the overall survival rate was 75% (50/67). The population was composed of 29 mares, 31 geldings and seven stallions. The breeds were Quarter horses (22), Thoroughbreds (8), Drafts (nine), Paints (seven), Arabians (five), Saddlebreds (five), Warmbloods (four), Miniatures (five), one Appaloosa, and one Standardbred. Thirty-six percent of horses required surgical intervention (24/67). Definitive diagnoses were available in the 24 surgical cases (Table 3). In 58% of the surgical cases (14/24) the primary lesion identified was localized to the large colon, 33% (8/24) were localized to the small intestine, and 8% (2/24) were localized to the small colon. There were no definitive diagnoses for the 43 non-surgical cases either because they responded to medical management or were subjected to humane euthanasia, and no necropsy was performed. Sixty seven percent of horses that were treated surgically survived to time of discharge (18/24).

**Table 3:** Location of primary lesion diagnosed at the time of surgery in retrospective and prospective populations.

**Location of primary lesion diagnosed at the time of surgery**	**Large colon**	**Small intestine**	**Small colon**
Surgical cases in retrospective population (*n* = 24)	58% (14/24)	33% (8/24)	8% (2/24)
Surgical cases in prospective population (*n* = 29)	66% (19/29)	31% (9/29)	3% (1/29)

### Univariate Logistic Regression

The 10 variables (heart rate, respiratory rate, total serum calcium concentration, blood and peritoneal fluid lactate concentration, band neutrophil count, fibrinogen and triglyceride concentration, abnormal ultrasound, and abnormal rectal exam) were analyzed in univariate logistic regressions to determine which variables provided the most accurate prediction of survival.

Univariate logistic regression analyses revealed six variables associated with survival in horses presenting for colic (Table 4). Probability of survival was decreased with an increase in heart rate, respiratory rate, blood lactate concentration, and presence of abnormal findings on rectal exam and abdominal ultrasound. Increased total calcium concentration was associated with higher odds for survival.

**Table 4:** Univariate logistic regression analysis for survival in the retrospective study.

**Variables**	***B***	**S.E**.	**Wald statistics**	**OR**	**95% CI**	***P***
Heart rate (bpm)	−0.3	0.15	5.34	0.96	0.94–0.99	0.02
Respiratory rate (bpm)	−0.73	0.03	6.16	0.93	0.87–0.98	0.01
Total Ca (mg/dL)	0.57	0.23	5.7	1.77	1.11–2.8	0.02
Lactate (mmol/L)	−0.27	0.12	5.3	0.75	0.6–0.95	0.02
Abnormal ultrasound exam	−1.3	0.63	4.4	0.26	0.07–0.9	0.04
Abnormal rectal exam	−3.8	1.9	3.9	0.02	0.01–0.9	0.04
Triglycerides (mg/dL)	−0.61	0.03	4.03	0.94	0.88–1.01	0.06
Peritoneal fluid lactate (mmol/L)	−0.27	0.12	5.3	0.76	0.6–1.12	0.055
Band neutrophil count	−0.57	0.35	2.64	0.56	0.28–1.12	0.1
Fibrinogen (mg/dL)	−0.04	0.02	5.12	0.99	0.9–0.98	0.06

*B, regression coefficient; S.E, standard error; OR, odds ratio; CI, confidence interval*.

### Receiver Operating Characteristic Curve in the Retrospective Study

**Figure 2:**
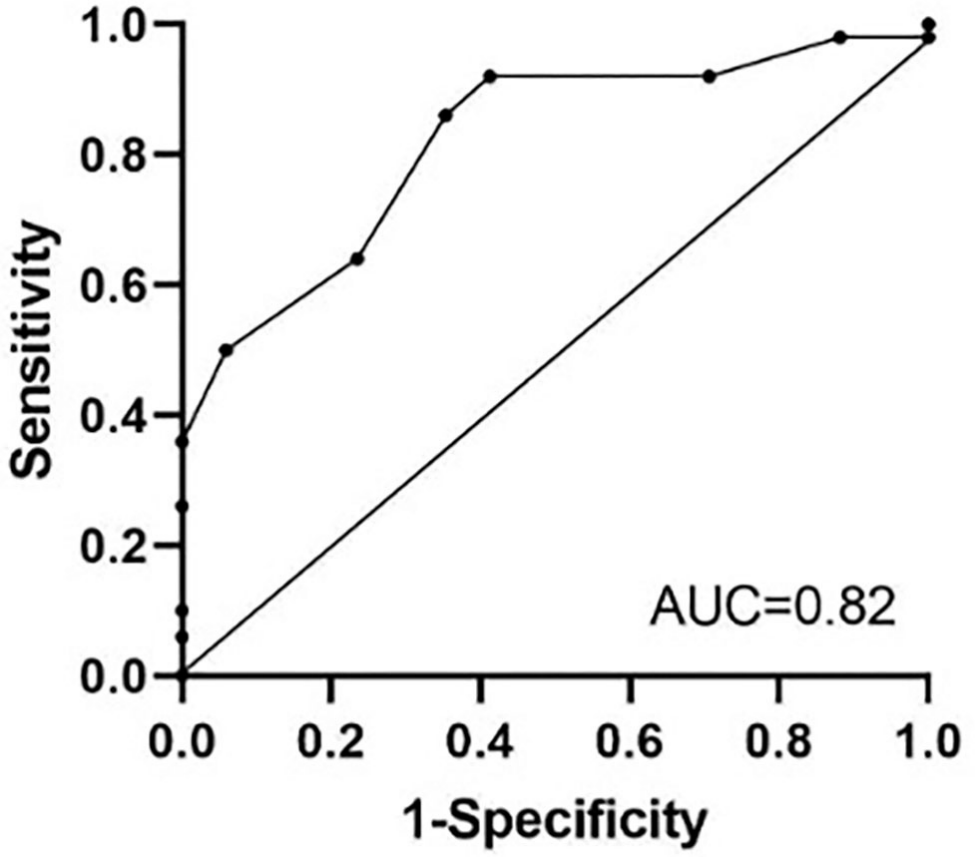
Receiver operating characteristics (ROC) curve for the colic assessment score to predict survival in the retrospective study. A cutoff value of 7 for the colic assessment score maximized sensitivity (86%) and specificity (64%) to predict survival in horses presenting for colic. AUC, area under the curve.

The receiver operating characteristic curve (ROC) for the CAS had an area under the curve of 0.82 (95% CI, 0.7–0.92) and indicated that a cutoff value of seven maximized sensitivity (86%, 95% CI, 77–93%) and specificity (64%, 95% CI, 39–89%) to predict survival in horses with abdominal pain (Figure 2). Positive (PPV) and negative predictive values (NPV) of the CAS to predict survival were 88 and 57%, respectively.

### Prospective Study

In the prospective study validating use of the CAS in a separate population of horses, the median age of horses was 12.5 years (7–28 years; interquartile range) and the survival rate was 76% (73/95). The population was composed of 40 mares, 48 geldings, and seven stallions. The breeds were Quarter horses (39), Arabians (14), Warmbloods (11), Paints (four), Missouri Fox Trotters (three), Thoroughbreds (three), Paso Finos (three), Miniatures (four), Morgans (three), Gypsy Vanners (two), one National Show Horse, one mule, and one Hackney. Thirty-one percent of horses required surgical intervention (29/95). Definitive diagnoses were available in the 29 surgical cases (Table 3). In 66% of the surgical cases (19/29) the primary lesion identified was localized to the large colon, 31% (9/29) were localized to the small intestine, and 3% (1/29) were localized to the small colon. Seventy-six percent of horses that were treated surgically survived to time of discharge (22/29). The ability of the CAS to predict survival was evaluated by the association of the final predicted outcome using a contingency table. Horses with a CAS > 7 were predicted to die and those with a CAS ≤ 7 were predicted to survive. This classification was compared to the actual outcome, and the sensitivity, and specificity of the CAS were 84% (95%CI, 76–92%) and 62% (95%CI, 44.4–79.6%), respectively. PPV and NPV of the CAS to predict survival were 88 and 52%, respectively. Positive likelihood ratio was 2.2 and negative likelihood ratio was 0.25.

### Univariate Logistic Regression for the Colic Assessment Score in the Prospective Study

Probability of survival decreased with a one-point increase in the CAS (OR = 0.69) (95% CI, 0.5–0.9) (Table 5).

**Table 5:** Univariate logistic regression for the colic assessment score to predict survival in the prospective study.

**Variables**	**B**	**S.E**.	**Wald Statistics**	**OR**	**95% CI**	**P**
Colic assessment score	−0.3	0.15	6.8	0.69	0.52–0.91	0.009

*B, regression coefficient; S.E, standard error; OR, odds ratio; CI, confidence interval*.

## Discussion

This study developed a colic assessment scoring system for use in horses that can help prognosticate survival in horses presenting for evaluation of colic. Further, results of the prospective portion of the study were valuable in validating this model for use in a clinical setting. This scoring system had an adequate sensitivity and positive predictive values (84 and 88%) with lower specificity and negative predictive value. The ideal scoring system should be based on routinely recordable variables, be applicable to all patient populations, and have a high level of discrimination between outcomes. The area under the ROC curve was used to test the ability of the CAS to differentiate between survival and non-survival and was determined to be 0.82. A model with the AUC of >0.7 is considered to have adequate discrimination (16). However, the wide 95% CI for AUC (0.7–0.92) limits the precision of the AUC as a test for the CAS discrimination. This scoring system should be assessed under local conditions so that its diagnostic ability is not overestimated.

The ability of this scoring system to predict mortality in colic cases should be evaluated relative to other scoring systems that have been reported. The sensitivity of the CAS (84%) is superior to that of the colic severity score (66.7%) reported by Furr in 1995, however the specificity of Furr's score (100%) was far superior to the specificity of the CAS (62%) (9). The predictive validity of the Equine Acute Abdominal Pain Scales in regards to mortality demonstrated 70% sensitivity and 71% specificity (11). The primary concern with the low specificity of the CAS is the likelihood of getting false positives (predicting non-survival in cases where the horse could survive). The variables assessed in the development the CAS were limited to the diagnostic findings available in the medical records, which can be limited in emergency scenarios such as colic exams. Inclusion of variables such as a pain score and peritoneal fluid lactate should be evaluated to assess if this improves the performance of the CAS in future studies.

The six variables in the CAS were chosen based on a strong predictive value for outcome at discharge. Several of the factors such as heart rate, respiratory rate and blood lactate have been recognized previously as significant predictors of death (4, 9, 10, 17–23). Abdominal ultrasound exam is routinely performed as an important diagnostic when evaluating a horse for colic signs. Ultrasound findings help to provide information on the type of lesion present within the abdomen (7, 13, 14). This is particularly valuable when evaluating for strangulating obstructions of the small intestine (13–15). Signs consistent with intestinal compromise or strangulation would include; increased volume of peritoneal fluid, alteration in echogenicity of peritoneal fluid, small intestinal wall thickness exceeding 3 mm, loss of intestinal motility, and progressive small intestinal distension (7, 13, 15). It may be valuable in future studies to evaluate specific ultrasound findings with survival to discharge.

Measurement of both venous blood lactate and peritoneal fluid lactate has been shown to be a valuable component of the diagnostic work-up for colic (19–24). L-lactate production is favored in hypoxic or anoxic conditions (19, 20). Colic lesions that result in bowel ischemia or endotoxemia create circumstances in which anaerobic glycolysis predominates as peripheral circulation collapses (19, 20). A previous study that focused on 360° volvulus of the ascending colon demonstrated that horses with plasma lactate concentrations <6.0 mmol/L at presentation had >90% chance of survival, whereas horses that had a plasma lactate >7.0 mmol/L had a 30% chance of survival (21). In our study, horses that had a peripheral venous lactate of >2.0 mmol/L received a score of 2 whereas horses that had peripheral lactate ≤2.0 mmol/L received a score of 0. This cutoff was selected based on reference plasma lactate values in healthy horses (25). Raising the cutoff value for plasma lactate would likely improve the specificity of the CAS. Inclusion of peritoneal fluid lactate in the CAS would most likely strengthen its predictive ability as well. Previous work has demonstrated the value of peritoneal lactate as a marker of ischemia and strangulating obstructions (24). This important parameter was not included in the CAS presented here due to a large percentage of horses that either did not have peritoneal fluid analysis at presentation or lack of documentation in the medical record.

Clinical pathologic values such as packed cell volume and venous blood lactate have been well-recognized as valuable prognostic indicators in patients presenting for abdominal pain. However, total serum calcium concentration has not been as extensively researched in its correlation with survival of colic (26–29). The relationship between total serum calcium and survival of patients may in part be explained by the finding that hypocalcemia can occur due to systemic endotoxemia and sepsis (26, 27). The pathogenesis of hypocalcemia in critically ill horses includes intracellular calcium sequestration, intestinal losses, parathyroid gland dysfunction and decreased intake (27). Hypocalcemia in horses with colic could be the result of intestinal losses, decreased intake or endotoxemia depending upon the type of lesion present and duration of clinical signs. Hypocalcemia and hypomagnesemia have been recognized in horses with strangulating small intestinal lesions during the perioperative period (28). Delesalle and colleagues found that hypocalcemia was recognized in 88% of horses that presented with an acute abdomen (29). They also reported a significant increase in the likelihood of development of intestinal hypomotility in horses that had hypocalcemia at presentation. The mechanism for hypocalcemia in our study is likely a combination of endotoxemia, decreased intake and intestinal losses. Total serum calcium was used in the development of the CAS rather than ionized calcium solely due to the retrospective nature of this study and the lack of availability of an ionized calcium in the medical records. Previous studies have demonstrated the prognostic value of ionized calcium in patients with gastrointestinal disease (29–31). The authors recognize that use of a total serum calcium value rather than the metabolically active, free ionized calcium is a limitation because it can be affected by albumin levels (27). Our findings support that calcium levels at presentation should be taken into consideration as a prognostic indicator in horses presenting for colic.

Several variables were not included in creation of the CAS due to lack of availability in the medical record. Pain scores were not included as a variable in the original 28 variables assessed in this study. Work has been done to help quantify the degree of pain in equine patients (11, 12, 23). Although pain scores have been shown to be helpful in creating a gravity score for prognosis in equine surgical colic (23), the retrospective nature of the study did not allow for inclusion of a pain score, because horses were not assigned a pain score during their initial exam. Abdominal auscultation is routinely performed during a colic exam, however this variable was also not included in the 28 variables assessed in our study. It was not included in the creation of the CAS due to lack of standardization in the medical records and questionable inter-observer reliability (32). Inclusion of abdominal auscultation in future studies with the CAS would be valuable so long as it was assigned a grade based on a previously published scoring system (33). Duration of colic signs is another important factor to consider when evaluating a horse for abdominal pain, but again this was not included in the CAS due to lack of availability in the retrospective data.

The retrospective nature of this study provided a myriad of limitations. The population of horses selected was limited to patients that had a complete colic exam (physical exam, abdominal ultrasound, abdominocentesis, and transrectal palpation) as well as a complete blood count and serum chemistry. The initial population was over 600 horses, but was ultimately reduced to 67 due to missing data. One concern expressed during collection of the data was the exclusion of colic patients that did not have a complete blood count and serum chemistry submitted at the time of presentation. Full bloodwork is not routinely evaluated in horses presenting for colic, which meant that a large number of horses were excluded in the retrospective portion of the study and therefore these numbers may not be the most representative of the “average” population of horses presented with colic. By including only cases with complete bloodwork it is possible that a more systemically compromised population was inadvertently selected for since blood work is more frequently submitted in cases where there is a history of fever, diarrhea, pneumonia, renal injury, or other comorbidities. It is also important to recognize that the number of non-survivors could have been affected by cognitive biases of the responsible clinician. The majority of non-survivors were euthanized (14/15), and this decision would have been based on the clinician's assessment, which is inherently susceptible to bias.

The inclusion of rectal and abdominal ultrasound findings in the CAS was important because therapeutic decisions made for clinical cases are often based on these findings. However, these findings are largely subjective in nature aside from mural thickness, or small intestinal diameter measurement, for example. Classifying rectal and ultrasound exams as simply “normal” or “abnormal” does not take into account the difference in prognosis for the variety of findings possible during these exams. This is one of the challenges in creating a simplified scoring system for something as broad as acute abdominal pain in the horse.

Finally, it would be interesting to assess the concurrent effect of several predictive factors on the survival in our study. Multivariable logistic regression allows evaluation of the simultaneous effect of multiple variables on the outcome, and it is a preferred method over univariate logistic regression (34, 35). Unfortunately, we were not able to apply this method due to the small number of non-survivors in our study and missing data for several variables.

In conclusion, the CAS developed in this study is applicable for clinicians in a hospital setting with a clinical caseload of horses with colic signs using data available in most equine practices. The CAS should be considered in light of the entirety of the clinical picture. Further evaluation and validation of this scoring system in a larger population of horses from multiple hospitals with the inclusion of ambulatory practice will strengthen its use in clinical practice.

## Data Availability Statement

The original contributions presented in the study are included in the article/supplementary material, further inquiries can be directed to the corresponding author/s.

## Ethics Statement

Ethical review and approval was not required for the animal study because all owners of horses undergoing clinical evaluation consented to the use of all case information and images for scientific publication. Written informed consent was obtained from the owners for the participation of their animals in this study.

## Author Contributions

AF and KD contributed to conception and design of the study. AF organized the database and wrote the first draft of the manuscript. KD performed the statistical analysis. All authors contributed to manuscript revision, read, and approved the submitted version.

## Conflict of Interest

The authors declare that the research was conducted in the absence of any commercial or financial relationships that could be construed as a potential conflict of interest.

## Publisher's Note

All claims expressed in this article are solely those of the authors and do not necessarily represent those of their affiliated organizations, or those of the publisher, the editors and the reviewers. Any product that may be evaluated in this article, or claim that may be made by its manufacturer, is not guaranteed or endorsed by the publisher.
